# Age at First Sexual Intercourse and Multiple Sexual Partnerships Among Women in Nigeria: A Cross-Sectional Analysis

**DOI:** 10.3389/fmed.2018.00171

**Published:** 2018-06-08

**Authors:** Sanni Yaya, Ghose Bishwajit

**Affiliations:** School of International Development and Global Studies, University of Ottawa, Ottawa, ON, Canada

**Keywords:** age at sexual debut, multiple sexual partnerships, global health, maternal health, women's health, Nigeria, demographic and health survey

## Abstract

**Background:** Little is known about sexual behavior such as first sexual intercourse and number of sexual partnerships among women in Nigeria. Early sexual debut is a widely recognized public health issue due to its influence on higher lifetime sexual partners which in turn is associated with increased vulnerability to pregnancy complications, HIV/AIDS and other and sexually-transmitted diseases. In the present study, we attempted to explore the patterns of age of sexual debut and multiple sexual partnerships among women of reproductive age in Nigeria.

**Methods:** Women who responded to the questions about the age at first sex and number of lifetime sex partners were selected from two latest rounds Nigeria Demographic and Health Survey (DHS). In total 60,611 women aged between 15 and 49 years were selected for this analysis. Age at sexual debut was used as the predictor of multiple sexual partnerships which was assessed by multinomial regression models with logit link function in complex sample analysis mode.

**Results:** The median age at first sex was 16 years (Interquartile range 16–24). Age at first sexual intercourse below the age of 19 years was reported by 30.8% of the women. Respectively 45.4% (95%CI = 42.9–47.9) 49.8% (95%CI = 47.8–51.7) of the women reported experiencing first intercourse before reaching 15 and 17 years, whereas 46.9% (95%CI = 45.2–48.7) of the women reported being monogamous and 47.2% (95%CI = 45.6–48.8) and 47.6% (95%CI = 43.8–51.3) had 2–3 and >3 lifetime sexual partners. In multivariable analysis after adjusting for confounding factors, women having sexual debut below 18 years were found to be significantly more likely to have 2–3 and more than 3 lifetime sexual partner.

**Conclusion:** The study concludes that an increasing proportion of Nigerian women are experiencing sexual debut before reaching 15 years. The findings suggest that early sexual debut is associated with multiple sexual partnerships which may increase the risk of STIs. Stakeholders in health care system need to be aware that early sexual debut can be associated with successive unsafe sexual practices which can lead to adverse health outcomes including HIV infection and STIs, early marriage, unwanted pregnancy, and abortion. Therefore, it is important to design effective interventions to encourage women delay sexual debut to help prevent unintended pregnancies and decrease the disproportionate burden of adverse health outcomes.

## Introduction

Transitions from childhood to adolescence and from adolescence to early adulthood represent two major life transition periods where individuals experience behavioral, cognitive and psychosocial changes (1, 2). Sexuality is one of the major changes that signify this transition characterized by a combination of anticipation and anxiety toward the first sexual experience (3). Along with these transformations, come the responsibilities of independent decision-making, exploring new relationships, and psychosexual directions, which expose the adolescents to a unique set of emotional and sexual vulnerabilities, especially in the marginalized and unsupportive environments (4). These exert long-term impact on their physical and psychological health and well-being, and ultimately influence their capacity to realize their full potential (5). Hence, understanding the patterns of risky sexual behavior and meeting the health needs remain key priorities for healthcare systems in developing countries.

A fall in the age at menarche, the onset of female sexual maturity and a similar shift in earlier ages of sexual maturity for males have been reported (6–8). The early maturity could be accompanied by a decreasing trend in the age at sexual debut. Adolescents who initiate sex at early ages are more likely than those who do not initiate sex early to experience risks of poor sexual and reproductive health outcomes such as abortions, STIs including HIV/AIDS, sexual abuse, unplanned and repeated pregnancies (9–11). Moreover, early age at sexual debut has also be shown to decrease condom use, women's sexual right and ability of abstinence (10), subsequently regretting non-consensual sexual acts (12, 13) which can significantly impair reproductive health and affect overall quality of life among women. While this changing trend is being felt in both high-income and low-and-middle income countries, the consequences are particularly worrisome for regions or countries with high rates of HIV/AIDS such as sub-Saharan Africa (SSA).

Nigeria, as the most populated country in African continent, has reported high burden of sexual health problems including HIV/AIDS (14), and has one of the highest adolescent birth rates in the world (15). About 28% of Nigerian adolescent are said to be sexually active with median age at sexual debut of 15 years, and an increasing proportion of those aged between 15 and 19 years engaging in sexual acts (16). Females are also reported to have a lower age of sexual debut than males and the main reasons for early sexual debut are “love,” peer pressure and rape (17). Girls are often more vulnerable to the risks of adverse consequences of early sexual debut due to poor women's empowerment such as inadequate decision making power, susceptibility to intimate partner violence (higher rate of reporting forced sexual initiation), lack of access to health information and economic support. Besides early sexual initiation, multiple sexual partnership (MSPs) has also been recognized as a significant predictor of STIs and lower use of condom (18, 19).

Individual's sexual behavior, including fertility preference and number of sexual relationships, is a complex construct which is shaped by values embedded within various familial, religion, cultural, and social contexts. For example, young people may choose to have multiple relationships in order to feel more confident and efficient in choosing life partner as they become mature (20). In Nigeria, secondary school students from polygamous family structure were found more likely to have MSPs, compared to those from monogamous family background (15). Adolescents who initiate sex at younger ages are more likely than those who do not, to have MSPs (20). Similarly, there can be broad range of pathways through which early sexual debut can lead to MSPs. This study aimed to measure the prevalence of early sexual debut and MSPs among women using data from two representative national surveys in Nigeria.

## Methods

### The survey and sampling design

DHS are nationally representative that collect information on a wide range of public health related topics such as anthropometric, demographic, socioeconomic, family planning, and domestic violence amongst others. The survey covered men and women aged between 15 and 49 years and under-5 children residing in non-institutional settings. NDHS 2008 and NDHS 2013 were the third and fourth round of DHS to be conducted in the country. In Nigeria, DHS field work is implemented by the National Population Commission (NPC) with the financial and technical assistance by ICF International provisioned through the USAID-funded MEASURE DHS program. For sampling, a three-staged stratified cluster design was employed which was based on a list enumeration areas (EAs) from the 2006 Population Census of the Federal Republic of Nigeria. EAs are systematically selected units from the localities, which constitute the local government areas (LGAs). LGAs are subdivisions of each of the 36 administrative states (including the Federal Capital Territory called Abuja) and classified under six developmental zones in the country. EAs were used to form the survey clusters called primary sampling units. A more detailed version of the survey was published elsewhere (21).

### Inclusion criteria

Main inclusion criteria were being 15–49 years of age regardless of current marital status. In total 60,611 observations from last two rounds of the surveys met these criteria (28,502 respondents from 2008 DHS, and 32,109 from 2013 DHS).

### Outcome and explanatory variables

The outcome variable was number of lifetime sexual partners. It was measured based on the responses to the question: total lifetime number of sexual partners. For analysis it was categorized as: 1, 2–3, and >3 partners.

The main explanatory variable was age at sexual debut, which was measured based on the responses to the question: age at first intercourse. The cut-off for early sexual debut ranges from <15 (22), <16 (23) to <18 years (23) in different studies. For this study, we measured the prevalence of both thresholds and categorized as: <15 years, 15–17 years, ≥18 years.

### Control variables

Besides the main explanatory variables, the following factors were likely to be correlated with age at sexual debut and number of sexual partners: Age groups (15–24/25–29/30–34/35–49), Region: North Central/North East/North West/South East/South South/South West, Residency (rural/urban) usually determined based on community population size, availability of basic amenities and predominant occupation, Religion (Christian/Islam/Other), Education (Nil/Primary/Secondary/Higher), DHS obtain wealth scores by principal components analysis, based on a list of household assets owned by respondents, which could include; type of floor, wall, roof, water supply, sanitation facilities, radio, electricity, television, refrigerator, cooking fuel, furniture, number of persons per room. Based on the weighted wealth scores, households are grouped into five wealth categories; poorest/poorer/middle/richer/richest.

### Statistical analysis

Data were analyzed using SPSS version 24. The dataset was screened for missing values and then merged to perform pooled analysis. Owing to the clustered structure of DHS data, we used complex survey design method for all analyses. The prevalence of those experienced first intercourse before 15, 15–17 years and at 18 years or above were described by percentages with 95%CIs. Same was done to represent the prevalence of lifetime sexual partners. The basic sociodemographic variables were also described alongside Chi-square bivariate tests to examine the group differences (age of sexual debut below 15, 15–17, and at least 18 years, and number of sexual partners 1, 2–3, >3). The variables that showed significance at *p* ≤ 0.25 in the bivariate tests were retained for final regression analysis. The *p* ≤ 0.25 was chosen to allow the inclusion of more explanatory variables in the regression model. However, the level of significance in the regression model was set at *p* < 0.05. The association between age of sexual debut and number of sexual partners was calculated by multinomial logistic regression. The first model included no other variables to measure the unadjusted odds ratios, while the second model controlled for the potentially confounding variables to produce the adjusted odds ratios.

### Ethical clearance

We did the analyses using publicly available data from demographic health surveys. Ethical procedures were the responsibility of the institutions that commissioned, funded, or managed the surveys. All DHS surveys are approved by ICF international as well as an Institutional Review Board (IRB) in respective country to ensure that the protocols are in compliance with the U.S. Department of Health and Human Services regulations for the protection of human subjects.

## Results

### Descriptive characteristics

Table 1 summarizes the basic sociodemographic characteristics of the sample population. Mean age of the respondents was 30.68 (9.0). Most of them were aged between 25 and 29 years (21.1%), from North West region (26.8%), rural residents (66.9%), followers of Islam (51%), and had no formal education (43.9%).

**Table 1:** Sample description.

			**Median age at first intercourse**		
	***N* = 60,611**	**%**	**2nd quartile**	**Median**	**4th quartile**
**YEAR**					
2008	28,502	47.0	14	16	20
2013	32,109	53.0	14	16	20
**AGE COHORTS (30.68**, ***SD*****: 9)**					
15–19	6,139	10.1	14	15	16
20–24	10,659	17.6	15	16	19
25–29	12,763	21.1	15	18	20
30–34	9,658	15.9	14	16	21
35–39	8,443	13.9	15	16	21
40–44	6,553	10.8	14	16	21
45–49	6,397	10.6	14	16	20
**REGION**					
North Central	10,212	16.8	15	18	22
North East	11,350	18.7	14	15	18
North West	16,214	26.8	14	15	16
South East	6,233	10.3	16	19	23
South-South	8,299	13.7	15	18	20
South West	8,304	13.7	16	19	21
**RESIDENCY**					
Urban	20,060	33.1	15	18	21
Rural	40,552	66.9	14	15	19
**RELIGION**					
Christian	28,535	47.1	15	18	21
Islam	30,926	51.0	14	15	18
Other	1,151	1.9	14	16	20
**EDUCATION**					
None	26,594	43.9	14	15	16
Primary	12,003	19.8	15	16	20
Secondary	16,648	27.5	15	18	20
Higher	5,366	8.9	18	20	24
**WEALTH INDEX**					
Poorest	13,381	22.1	13	15	17
Poorer	12,921	21.3	13	15	18
Middle	11,780	19.4	13	16	18
Richer	11,568	19.1	13	16	19
Richest	10,961	18.1	14	18	21

*NDHS 2003–2013*.

Median age at sexual debut was 16 years, which tended be lower among those currently aged below 19 years, located in the North East and North West region, rural residents, followers of Islam, had no formal education, and from households with lower wealth status.

### Early sexual debut

As shown in Table 2, the prevalence of those having first sexual intercourse before reaching 15 years, 15–17 years, and after 17 years was respectively 26.6% (25.8–27.4), 29.2% (28.6–29.8), and 44.2% (43.3–45.2); The likelihood of first intercourse before 15 years and between 15 and 17 years were significantly higher among those, located in the North West region, living in rural areas, followers of Islam and had no formal education. In contrast those who experienced first intercourse at 18 and above were more likely to be from North Central region, followers of Christianity and had secondary level education. Prevalence of sexual debut before reaching 15 years were 45.4% (95%CI = 42.9–47.9) and that of between 15 and 17 years was 49.8% (95%CI = 47.8–51.7).

**Table 2:** Age at sexual debut.

**Variable**	**<15 years (*n* = 16,108)**	**15–17 years (*n* = 17,696)**	**≥18 years (*n* = 26,810)**
	**26.6% (25.8–27.4)**	**29.2% (28.6–29.8)**	**44.2% (43.3–45.2)**
**YEAR**			
2008	45.4, 42.9, 47.9	49.8, 47.8, 51.7	46.2, 44.7, 47.7
2013	54.6, 52.1, 57.1	50.2, 48.3, 52.2	53.8, 52.3, 55.3
*P*	<0.001		
**AGE**			
15–19	14.6, 13.8, 15.4	17.4, 16.7, 18.2	2.7, 2.4, 2.9
20–24	15.5, 14.8, 16.2	18.4, 17.7, 19.1	18.3, 17.7, 18.9
25–29	18.6, 17.9, 19.3	18.8, 18.1, 19.5	24, 23.4, 24.7
30–34	15.2, 14.6, 15.9	13.9, 13.3, 14.6	17.7, 17.1, 18.2
35–39	12.9, 12.2, 13.6	13.2, 12.6, 13.9	15, 14.5, 15.5
40–44	11.2, 10.6, 11.8	9.2, 8.7, 9.8	11.6, 11.1, 12.1
45–49	12, 11.4, 12.8	9, 8.5, 9.6	10.7, 10.2, 11.2
*P*	<0.001	
**REGION**			
North Central	10.2, 9.4, 11.1	14.5, 13.5, 15.6	22.4, 21.5, 23.4
North East	25.8, 23.9, 27.9	22.8, 21.3, 24.3	11.8, 10.8, 12.8
North West	45.6, 43.3, 48	32, 30.2, 33.8	12, 11.1, 12.9
South East	4.3, 3.8, 4.8	6.7, 6.1, 7.3	16.3, 15.3, 17.3
South-South	9.4, 8.6, 10.3	14.6, 13.6, 15.6	15.7, 14.7, 16.7
South West	4.6, 4.1, 5.2	9.5, 8.8, 10.3	21.9, 20.8, 23.1
*P*	<0.001	
**RESIDENCY**			
Urban	19.3, 17.5, 21.3	25.3, 23.7, 26.9	46.5, 45, 48
Rural	80.7, 78.7, 82.5	74.7, 73.1, 76.3	53.5, 52, 55
*P*	<0.001	
**RELIGION**			
Christian	25.5, 23.8, 27.3	40.3, 38.3, 42.2	64.5, 62.9, 66.1
Islam	72.4, 70.5, 74.2	58, 56, 60	33.6, 32, 35.2
Other	2.1, 1.7, 2.6	1.7, 1.4, 2.1	1.9, 1.6, 2.3
*P*	<0.001	
**EDUCATION**			
None	68.5, 66.8, 70.1	51.5, 49.8, 53.3	24.1, 22.8, 25.3
Primary	17.8, 16.7, 18.9	21.3, 20.3, 22.4	20, 19.2, 20.9
Secondary	12.6, 11.7, 13.5	24.2, 23.1, 25.4	38.6, 37.5, 39.7
Higher	1.2, 1.0, 1.4	2.9, 2.6, 3.3	17.4, 16.4, 18.4
*P*	<0.001	
**WEALTH INDEX**			
Poorest	43.4, 41.6, 45.2	35.0, 33.5, 36.5	21.6, 20.2, 23.1
Poorer	35.4, 34.0, 36.9	34.4, 33.2, 35.6	30.2, 28.7, 31.7
Middle	24.3, 23.1, 25.5	31.1, 30.0, 32.3	44.6, 43.1, 46.1
Richer	16.5, 15.4, 17.5	26.6, 25.5, 27.6	57.0, 55.5, 58.4
Richest	8.7, 8.0, 9.5	16.6, 15.7, 17.6	74.6, 73.3, 75.9
*P*	<0.001	

*N.B. p-values (Pearson's Chi-square) indicate the probability of observing statistically significant relationships between column and row variables*.

### Lifetime of sexual partners

Results from Table 3 showed that having 1, 2–3, and >3 lifetime sexual partners were 67.8% (67.0–68.6), 27.8% (27.2–28.5), and 4.3% (4.0–4.7), respectively. Regarding the number of lifetimes sexual partners, those who were monogamous were more likely to be from North West, followers of Islam, had secondary level education, and lived in poorest to poorer households. While the prevalence of having 2–3 and more than three were highest among those aged between 25 and 29 years.

**Table 3:** Number of lifetime partners.

**Variable**	**Monogamous (*n* = 41,110)**	**2-3 partners (*n* = 16,875)**	**>3 partners (*n* = 2,626)**
	**67.8% (67.0–68.6)**	**27.8% (27.2–28.5)**	**4.3% (4.0–4.7)**
**YEAR**			
2008	46.9, 45.2, 48.7	47.2, 45.6, 48.8	47.6, 43.8, 51.3
2013	53.1, 51.3, 54.8	52.8, 51.2, 54.4	52.4, 48.7, 56.2
*P*	<0.001	
**AGE**			
15–19	12.5, 12, 13	5.2, 4.7, 5.7	4.5, 3.6, 5.5
20–24	18.8, 18.3, 19.3	15.2, 14.5, 15.9	14.1, 12.6, 15.8
25–29	20.5, 20, 20.9	22.1, 21.3, 22.9	23.9, 22.1, 25.8
30–34	14.8, 14.4, 15.2	18.1, 17.4, 18.9	19.8, 18, 21.6
35–39	13, 12.6, 13.5	15.6, 15, 16.3	17.3, 15.5, 19.2
40–44	10.2, 9.8, 10.5	12.4, 11.8, 13	10.7, 9.4, 12.2
45–49	10.2, 9.8, 10.7	11.4, 10.8, 12.1	9.7, 8.4, 11.2
*P*	<0.001	
**REGION**			
North Central	18.1, 17.3, 18.9	14, 13.1, 15	15.7, 12.7, 19.3
North East	20.5, 19.1, 21.9	15.3, 14.2, 16.4	13.7, 11.1, 16.8
North West	33.3, 31.7, 34.9	14.3, 13.2, 15.5	3.8, 3, 4.9
South East	9.2, 8.5, 9.8	12.6, 11.6, 13.6	12.9, 10.9, 15.2
South-South	8.8, 8.2, 9.4	22, 20.8, 23.2	37.2, 33.8, 40.8
South West	10.2, 9.5, 10.9	21.8, 20.5, 23	16.7, 14.4, 19.3
*P*	<0.001	
**RESIDENCY**			
Urban	29.6, 28, 31.2	40.3, 38.8, 41.9	41.3, 37.8, 44.9
Rural	70.4, 68.8, 72	59.7, 58.1, 61.2	58.7, 55.1, 62.2
*P*	<0.001	
**RELIGION**			
Christian	37.7, 36, 39.4	63.9, 62.2, 65.6	86, 83.9, 88
Islam	60.3, 58.6, 62.1	34.4, 32.7, 36.2	11.9, 10.1, 13.9
Other	2, 1.6, 2.4	1.7, 1.4, 2	2.1, 1.3, 3.3
*P*	<0.001	
**EDUCATION**			
None	51.8, 50.2, 53.4	29, 27.7, 30.5	15.1, 13, 17.4
Primary	18.6, 17.8, 19.4	22.1, 21.2, 23.1	24.1, 22, 26.4
Secondary	22.9, 22, 23.9	36.2, 35, 37.4	42.8, 40.1, 45.4
Higher	6.7, 6.2, 7.2	12.7, 11.8, 13.6	18, 15.9, 20.3
*P*	<0.001	
**WEALTH INDEX**			
Poorest	79.2, 77.6, 80.7	18.5, 17.4, 19.8	2.3, 1.8, 2.9
Poorer	74.9, 73.5, 76.3	22.2, 21.0, 23.4	2.9, 2.5, 3.4
Middle	66.7, 65.2, 68.1	28.6, 27.4, 29.9	4.7, 4.2, 5.3
Richer	60.7, 59.3, 62.2	33.8, 32.6, 35.1	5.4, 4.9, 6.1
Richest	54.3, 52.7, 55.9	38.7, 37.3, 40.1	7.0, 6.3, 7.7
*P*	<0.001	

*N.B. p-values (Pearson's Chi-square) indicate the probability of observing statistically significant relationships between column and row variables*.

Table 4 contains the results of the association between age at sexual debut and number of lifetime sexual partners. It shows that those who had first intercourse before reaching 15 years and between 15 and 17 years had higher odds of having 2–3 and more than 3 lifetimes sexual partners. In the second model after adjusting for all potential confounding variables, compared with those who had first sex after age 18 years, the odds of having 2–3 lifetime sexual partners were 1.4 times higher among those who had first sex before reaching 15, and 1.5 times higher among those who had first sex before 18 (between 15 and 17 years). The corresponding odds of having >3 were respective lifetimes partners were 1.3 and 1.7 times higher among those who had first intercourse before 15 and before 18.

**Table 4:** Multinomial logistic regression on the association between early sexual debut and number of lifetime sexual partners.

**Variable**	**2–3 partners**		>**3 partners**		**2–3 partners**		>**3 partners**	
	**Crude 95%CI**	***P***	**Crude 95%CI**	***P***	**Adjusted 95%CI**	***P***	**Adjusted 95%CI**	***P***
**AGE AT SEXUAL DEBUT**								
18	1		1		1		1	
15–17	1.85(1.21–4.89)	<0.001	2.78(2.52–3.07)	<0.001	1.45(1.38–1.52)	<0.001	1.26(1.09–1.82)	<0.001
<15	1.72(1.29–2.57)	<0.001	2.76(2.46–3.10)	<0.001	1.50(1.42–1.58)	<0.001	1.68(1.21–2.87)	<0.001

*OR, odds ratios; CI, confidence interval. Reference group of number of sexual partners = Monogamous. Model adjusted for age, region, place of residence, religion, education, wealth index*.

## Discussion

### Main findings

Based on the analysis of Nigeria Demographic and Health Survey, the findings of the study suggest a considerably strong relationship between early sexual debut and lifetime experience of having multiple sexual partners. More than half of the respondents reported experiencing sexual debut before reaching 18 years, among which more than quarter reported same before becoming 15 years. Median age at sexual debut was 16 with significant disparities among different socioeconomic and regional groups. For instance, the percentage of women who had experienced first intercourse before 15 years and between 15 and 17 years were significantly higher among those living in the Northern region compared with those in the Southern region. This difference might be explained by the educational, economic, and healthcare disparities between these regions. Inhabitants in the Southern part of the country usually enjoy better socioeconomic prosperity and better healthcare facilities. The relationship between sexual debut, fertility, and educational status is a bidirectional one. While getting married or becoming pregnant usually mark the end of school life among adolescent girls in most societies in Africa, not being a school-goer can also enhance the possibility of early marriage/sexual experience. Evidence of early marriage among girls who are poor, have low education and living in rural areas are available from Asian settings (24). Thus, it is arguable that higher rates of early sexual debut and having multiple sexual partners in the Northern region are reflection of the socioeconomic disparities in comparison with the Southern. These factors should be taken into consideration when designing intervention programs.

Significant differences in the age at sexual debut and MSPs were observed for religious groups as well. The relationship between religion and individual's sexual behavior is not straightforward, and may vary substantially across gender, family structure, level of education. However, there are empirical evidences on the positive role religious beliefs delayed onset of sexual intercourse among adolescents (25, 26). These findings imply the role that religious institutions can play in influencing the collective standards and values that direct individual sexual behavior. There are several religious teachings that stand against certain medical practices such as abortion and contraceptive use, and this could prevent women in having sexual affairs capable of leading to pregnancy outside legal union including marriage. Christianity movement, for instance, has promoted the dissemination of clear doctrines and standards, along with disciplinary sanctions, especially to the sexuality of its followers in regard to fornication. Besides, religious groups have used various resources to create room to encourage young followers to participate in a religious environment. Dating groups, youth group, counseling services, and other activities have frequently been established in religious groups, which may have been successful at creating channels through which religion can influence the lives and behavior of young people and adolescents.

### Previous studies

Comparison of early sexual debut from previous studies is tricky, as the definition of early age seem to vary across regions. In a recent study on secondary school students in Ido-ekiti, of South-West Nigeria found that the mean age of sexual debut was 13.10 with more than two-third of the students had early sexual debut (27). In contrast with the prevalence of sexual debut of 26.6% (<15 years) and 29.2% (<17 years) in our study, the rates were lower than in Zimbabwe (11.8% before reaching 18 years) (28), but higher than in Malawi (29.6% before reaching 16 years), South Africa (39% before reaching 16 years) (29), and Tanzania (57.8% before reaching 15 years) (30). Our finding on the association between age at sexual debut and having multiple sexual partners among women is in line with several previously published studies. Multiple Indicator Cluster Survey study on Vietnam found significant association between early sexual debut with having lifetime multiple sexual partners (31). The same study also reported that first sexual intercourse before 19 years increased the likelihood of having multiple sexual partners by a factor of five. In south Africa, MSP was found to be significantly more common among men and women who reported early sexual debut compared with those who had late sexual debut (29). In the U.S., a longitudinal study on minority adolescents also reported that intercourse at an early age significantly increased the likelihood of high risk behaviors including having MSPs (32).

### General discussion and policy recommendations

Findings of this study on Nigeria support the findings from other countries in Africa regarding the positive the relationship between age at sexual debut and MSPs. The findings underscore the importance of taking programmatic measures to discourage early sexual debut among women. Currently there is insufficient evidence on sexual behavior among men and women in Nigeria. The country has in place since 2000 a national health policy to preventing adolescent behavioral risk factors of STIs, including HIV, associated adolescents. Despite that, developing intervention programs have been hampered by inadequate information on contextual factors associated with sexual behavior of adolescents (15). There is a need for more in-depth researches to explore the underlying cultural and sociodemographic factors that might be contributing to early sexual intercourse in Nigeria. Given the high prevalence rates of HIV/AIDS in the country, policy makers should invest on developing strategies to prevent of risky sexual behavior as an effort to reduce the risk of transmission. Promoting school-enrolment among adolescent girls can prove to be highly rewarding in this respect. Special attention should be given to adolescent girls contribute to a large proportion of all clandestine abortions (10) and HIV sero-prevalence in the country (14).

### Strengths and limitations

We have several important limitations to declare. Firstly, this was a cross-sectional study which means that the associations cannot guarantee any causal relationship. The datasets were secondary; therefore, we had no control over the selection and measurement of variables. It is very likely that some important variables were not adjusted for, especially those relevant to sociocultural values. The analysis includes only women, therefore the findings cannot provide the scenario for the general population. Lastly, the possibility of recall bias cannot be ignored as most of the respondents were above adolescent age. These limitations notwithstanding, this study provides important insights on sexual behavior among Nigerian women. This is the first country representative study to show the association between age at sexual debut and number of life times sexual partners among Nigerian women. Sample size was large and findings are generalizable for women aged between 15 and 49 years. As previous studies used different thresholds for early sexual debut, we used two different cut-offs instead of one.

## Conclusions

In conclusion, the findings of the present study revealed that an increasing proportion of Nigerian women are experiencing sexual debut before reaching 15 years, and that women who experienced early initiation are more likely to have multiple sexual partners. More research needs to be undertaken to understand the risk factors of risky sexual behavior among men and women in Nigeria. More so, National programs aimed at controlling early sexual debut and multiple sexual partners should emphasize on promoting comprehensive sexual health education. The findings suggest that early sexual debut is associated with multiple sexual partnerships which may increase the risk of unsafe sexual practices such as STIs and HIV/AIDS. Early sexual debut among women has become an emerging global concern since it exposes women to a myriad of reproductive health issues such as unwanted pregnancy, early marriages, abortions, sexually transmitted, and STIs. Women could engage in early sexual initiation basically due to poverty, myth and socio-cultural misconceptions, curiosity, and experimenting, absence of duty bearers including religious leaders, parents, teachers, aunts, and uncles in the sexuality socialization process. In addition, coercion, media, cultural initiation ceremonies, peer, and sibling pressure could also increase the occurrence of early sexual debut. Therefore, this study recommends active participation and integration of all duty bearers in the socialization process especially of the girl-child and enforcement of reproductive health and behavior laws in order to reduce the prevalence of early sexual debut.

## Availability of data and materials

Data for this study were sourced from Nigerian Demographic and Health surveys (DHS) and available here: https://dhsprogram.com/data/available-datasets.cfm.

## Author contributions

SY and GB contributed to the study design, the review of literature, and analysis of literature, manuscript conceptualization and preparation. They both critically reviewed the manuscript for its intellectual content. SY had final responsibility to submit for publication. All authors read and approved the final manuscript.

### Conflict of interest statement

The authors declare that the research was conducted in the absence of any commercial or financial relationships that could be construed as a potential conflict of interest.

## Abbreviations

DHS: Demographic Health Survey
IRB: Institutional Review Board
HIV/AIDS: Human immunodeficiency virus infection and acquired immune deficiency syndrome
SSA: Sub-Saharan Africa
LGAs: Local government areas
MSPs: Multiple sexual partnership.

## References

[1] Tulloch T Kaufman M Adolescent sexuality. Pediatr Rev. (2013) 34:29–38. 10.1542/pir.34-1-29 23281360

[2] Lenz B. The transition from adolescence to young adulthood: a theoretical perspective. J Sch Nurs. (2001) 17:300–6. 10.1177/10598405010170060401 11804406

[3] Olesen TB Jensen KE Nygård M Tryggvadottir L Spare'n P Hansen BT Young age at first intercourse and risk-taking behaviours—a study of nearly 65 000 women in four Nordic countries. Eur J Public Health (2012) 22:220–4. 10.1093/eurpub/ckr055 21596800

[4] Gambadauro P Carli V Hadlaczky G Sarchiapone M Apter A Balazs J. Correlates of sexual initiation among European adolescents. PLoS ONE (2018) 13:e0191451. 10.1371/journal.pone.0191451 29420612 PMC5805230

[5] Vasilenko SA Lefkowitz ES Welsh DP Is sexual behavior healthy for adolescents? A conceptual framework for research on adolescent sexual behavior and physical, mental, and social health. New Dir Child Adolesc Dev. (2014) 144:3–19. 10.1002/cad.20057 24962359

[6] Goldstein JR. A secular trend toward earlier male sexual maturity: evidence from shifting ages of male young adult mortality. PLoS ONE (2011) 6:e14826. 10.1371/journal.pone.0014826 21857893 PMC3157338

[7] Bodzsar EB Studies on sexual maturation of Hungarian children. Acta Biol Szegediensis (2000) 44:155–65. Available online at: http://citeseerx.ist.psu.edu/viewdoc/download?doi=10.1.1.557.1448&rep=rep1&type=pdf

[8] Khadilkar VV Stanhope RG. Secular trends in puberty. Indian Pediatr. (2006) 43:475–8. 16820656

[9] Falb KL Annan J Kpebo D Cole H Willie T Xuan Z. Differential impacts of an intimate partner violence prevention program based on child marriage status in Rural Côte d'Ivoire. J Adolesc Health (2015) 57:553–8. 10.1016/j.jadohealth.2015.08.001 26372368 PMC5783193

[10] Clark S. Early marriage and HIV risks in sub-Saharan Africa. Stud Fam Plann (2004) 35:149–60. 10.1111/j.1728-4465.2004.00019.x 15511059

[11] Raj A. When the mother is a child: the impact of child marriage on the health and human rights of girls. Arch Dis Child. (2010) 95:931–5. 10.1136/adc.2009.178707 20930011

[12] Wellings K Collumbien M Slaymaker E Singh S Hodges Z Patel D. Sexual behaviour in context: a global perspective. Lancet (2006) 368:1706–28. 10.1016/S0140-6736(06)69479-8 17098090

[13] Wight D Henderson M Raab G Abraham C Buston K Scott S. Extent of regretted sexual intercourse among young teenagers in Scotland: a cross sectional survey. BMJ (2000) 320:1243–4. 10.1136/bmj.320.7244.1243 10797033 PMC27366

[14] Fatusi AO Blum RW. Predictors of early sexual initiation among a nationally representative sample of Nigerian adolescents. BMC Public Health (2008) 8:136. 10.1186/1471-2458-8-136 18439236 PMC2390536

[15] Slap GB Lot L Huang B Daniyam CA Zink TM Succop PA. Sexual behaviour of adolescents in Nigeria: cross sectional survey of secondary school students. BMJ (2003) 326:15. 10.1136/bmj.326.7379.15 12511453 PMC139494

[16] Envuladu EA de Kwaak AV Zwanikken P Zoakah AI Exploring the factors influencing adolescent sexual behavior in plateau state Nigeria. Am J Med Med Sci. (2017) 7:1–6. 10.5923/j.ajmms.20170701.01

[17] Folayan MO Odetoyinbo M Brown B Harrison A. Differences in sexual behaviour and sexual practices of adolescents in Nigeria based on sex and self-reported HIV status. Reprod Health (2014) 11:83. 10.1186/1742-4755-11-83 25481734 PMC4266967

[18] Exavery A Lutambi AM Mubyazi GM Kweka K Mbaruku G Masanja H. Multiple sexual partners and condom use among 10 - 19 year-olds in four districts in Tanzania: what do we learn? BMC Public Health (2011) 11:490. 10.1186/1471-2458-11-490 21696581 PMC3141458

[19] Finer LB Darroch JE Singh S. Sexual partnership patterns as a behavioral risk factor for sexually transmitted diseases. Guttmacher Inst. (1999) 31:228–36. 10.1363/3122899 10723647

[20] Wilson Chialepeh N Sathiyasusuman A Associated risk factors of stis and multiple sexual relationships among youths in Malawi. PLOS ONE (2015) 10:e0134286 10.1371/journal.pone.0134286 26248328 PMC4527764

[21] NPC/Nigeria NPC-International ICF Nigeria Demographic and Health Survey 2013 (2014). Available online at: http://dhsprogram.com/publications/publication-fr293-dhs-final-reports.cfm

[22] Peltzer K Pengpid S. Early sexual debut and associated factors among in-school adolescents in six Caribbean Countries. West Indian Med J. (2015) 64:351–6. 10.7727/wimj.2014.025 26624586 PMC4909066

[23] Marston M Beguy D Kabiru C Cleland J. Predictors of sexual debut among young adolescents in Nairobi's informal settlements. Int Perspect Sex Reprod Health (2013) 39:22–31. 10.1363/3902213 23584465 PMC4101799

[24] Montazeri S Gharacheh M Mohammadi N Alaghband Rad J Eftekhar Ardabili H. Determinants of early marriage from married girls' perspectives in iranian setting: a qualitative study. J Environ Public Health (2016) 2016:8615929. 10.1155/2016/8615929 27123012 PMC4829716

[25] Lammers C Ireland M Resnick M Blum R. Influences on adolescents' decision to postpone onset of sexual intercourse: a survival analysis of virginity among youths aged 13 to 18 years. J. Adolesc. Health (2000) 26:42–8. 10.1016/S1054-139X(99)00041-5 10638717

[26] Rostosky SS Regnerus MD Wright MLC. Coital debut: the role of religiosity and sex attitudes in the Add Health Survey. J Sex Res. (2003) 40:358–67. 10.1080/00224490209552202 14735410

[27] Durowade KA Babatunde OA Omokanye LO Elegbede OE Ayodele LM Adewoye1 KR. Early sexual debut: prevalence and risk factors among secondary school students in Ido-ekiti, Ekiti state, South-West Nigeria. Afr Health Sci. (2017) 17:614–22. 10.4314/ahs.v17i3.3 29085388 PMC5656187

[28] Pettifor AE van der Straten A Dunbar MS Shiboski SC Padian NS. Early age of first sex: a risk factor for HIV infection among women in Zimbabwe. AIDS (2004) 18:1435–42. 10.1097/01.aids.0000131338.61042.b8 15199320

[29] Zuma K Mzolo T Makonko E. Determinants of age at sexual debut and associated risks among South African youths. Afr J AIDS Res. (2011) 10:189–94. 10.2989/16085906.2011.626283 25859787

[30] Mmbaga EJ Leonard F Leyna GH. Incidence and predictors of Adolescent's early sexual debut after three decades of HIV interventions in Tanzania: a time to debut analysis. PLoS ONE (2012) 7:e41700. 10.1371/journal.pone.0041700 22848571 PMC3407234

[31] Son DT Oh J Heo J Van Huy N Van Minh H Choi S. Early sexual initiation and multiple sexual partners among Vietnamese women: analysis from the Multiple Indicator Cluster Survey, 2011. Glob Health Action (2016) 9:29575. 10.3402/gha.v9.29575 26950566 PMC4780093

[32] O'Donnell L O'Donnell CR Stueve A. Early sexual initiation and subsequent sex-related risks among urban minority youth: the reach for health study. Fam Plann Perspect. (2001) 33:268–75. 10.1363/3326801 11804436

